# Arf6 is necessary for *senseless* expression in response to wingless signalling during *Drosophila* wing development

**DOI:** 10.1242/bio.058892

**Published:** 2021-12-02

**Authors:** Julien Marcetteau, Tamàs Matusek, Frédéric Luton, Pascal P. Thérond

**Affiliations:** 1 Université Côte d'Azur; UMR7277 CNRS; Inserm 1091; Institut de Biologie de Valrose (iBV); Parc Valrose, 06108 Nice cedex 2, Nice, France; 2 Université Côte d'Azur; UMR7275 CNRS; Institut de Pharmacologie Moléculaire et Cellulaire (IPMC), 660 Route des Lucioles, Sophia Antipolis, 06560 Valbonne, France

**Keywords:** *Drosophila*, Signalling, Wnt, Wingless, Arf6, Armadillo, Pangolin

## Abstract

Wnt signalling is a core pathway involved in a wide range of developmental processes throughout the metazoa. *In vitro* studies have suggested that the small GTP binding protein Arf6 regulates upstream steps of Wnt transduction, by promoting the phosphorylation of the Wnt co-receptor, LRP6, and the release of β-catenin from the adherens junctions. To assess the relevance of these previous findings *in vivo*, we analysed the consequence of the absence of Arf6 activity on *Drosophila* wing patterning, a developmental model of Wnt/Wingless signalling. We observed a dominant loss of wing margin bristles and Senseless expression in *Arf6* mutant flies, phenotypes characteristic of a defect in high level Wingless signalling. In contrast to previous findings, we show that Arf6 is required downstream of Armadillo/β-catenin stabilisation in Wingless signal transduction. Our data suggest that Arf6 modulates the activity of a downstream nuclear regulator of Pangolin activity in order to control the induction of high level Wingless signalling. Our findings represent a novel regulatory role for Arf6 in Wingless signalling.

## Abstract (precis)

**Summary:** Arf6 is necessary for the patterning of the wing margin by Wingless in *Drosophila melanogaster*, acting downstream of stabilized Armadillo but upstream or at the level of Pangolin activity.

## INTRODUCTION

The ADP-ribosylation factor (Arf) family of small GTP-binding proteins is remarkably well conserved throughout the eukaryotes (Donaldson and Jackson, 2011). Arf6 is the most divergent of the Arfs, and localises to the plasma membrane and endosomes where it regulates various steps of endosomal trafficking and recycling (D'Souza-Schorey and Chavrier, 2006; Donaldson and Jackson, 2011). Previous *in vitro* studies have implicated Arf6 in the upstream stages of Wnt signalling (Grossmann et al., 2013; Kim et al., 2013; Pellon-Cardenas et al., 2013). However, a potential physiological, *in vivo*, role of Arf6 in Wnt signalling is yet to be addressed (Kim et al., 2013).

Despite the evolutionary distance between humans and *Drosophila*, Arf6 shares 97% sequence identity conservation between the two species (Fig. S1A). Combined with the availability of powerful genetic tools, this makes *Drosophila* an ideal model in which to investigate the requirement for Arf6 in Wnt signalling in an *in vivo* context.

The *Drosophila* Wnt1 homologue, *wingless* (*wg*), is initially expressed throughout the wing primordium, and becomes progressively refined to a narrow strip of cells of the presumptive wing margin late in larval development (Ng et al., 1996; Williams et al., 1993). The *Drosophila* wing has classically served as a developmental model of Wg signalling and has played a fundamental role in our understanding of Wnt/Wg signalling (Bejsovec, 2018; Jenny and Basler, 2014; Langton et al., 2016; Wiese et al., 2018). Canonical Wg signalling is contingent upon the stability of cytoplasmic Armadillo (Arm, the *Drosophila* β-catenin homologue) in signal receiving cells. In the absence of the Wg ligand, Arm is constitutively phosphorylated by the β-catenin destruction complex, consisting of the scaffold Axin, APC, and the kinases GSK3β and CK1 (Stamos and Weis, 2013), promoting Arm proteasomal degradation. The binding of Wg to the Frizzled 2 (Fz2) receptor and Arrow (Arr) co-receptor at the cell surface activates Dishevelled (Dsh), leading to the deactivation of the destruction complex and the stabilisation of cytoplasmic Arm (Swarup and Verheyen, 2012). Arm then translocates to the nucleus where it binds to Pangolin (Pan, a LEF/TCF homologue), converting it from a transcriptional repressor to an activator, and triggering the expression of Wg target genes (Mosimann et al., 2009; Schweizer et al., 2003).

High level Wg signalling is essential for the establishment and patterning of the wing margin (Couso et al., 1994; Jafar-Nejad et al., 2006; Phillips and Whittle, 1993). Cells flanking the wing margin respond to the local high levels of Wg protein by expressing the zinc finger transcription factor *senseless* (*sens*), which acts as the proneural factor for the anterior stout mechanosensory, and posterior non-innervated margin bristles (Jafar-Nejad et al., 2003, 2006; Nolo et al., 2000). Low level Wg signalling further into the wing blade induces the expression of more sensitive target genes such as *distal-less* (*dll*), which is more broadly expressed in the wing blade (Neumann and Cohen, 1997; Zecca et al., 1996).

In this study we assessed the *in vivo* developmental role of Arf6 in Wg signalling using a *Drosophila* model. *Arf6* mutants show a dominant loss of wing margin bristles and a concomitant loss of Wg-dependent *sens* expression in the wing imaginal disc, phenotypes indicative of a defect in high level Wg signalling. Arf6 has previously been suggested to act upstream in the transduction of Wnt signalling by promoting the phosphorylation of the Wnt co-receptor, LRP6, and the release of β-catenin from the adherens junction into the cytoplasm (Grossmann et al., 2013; Kim et al., 2013; Pellon-Cardenas et al., 2013). In contrast to these findings, our data indicate that in *Drosophila* Arf6 is necessary downstream of Arm stabilisation for the activation of high level Wg signalling. Moreover, we show that Arf6 acts genetically upstream, or at the level of Pan activity. These findings represent a novel function for Arf6 necessary for high level Wg target gene expression during wing margin development, and is the first demonstration of an *in vivo* role for Arf6 in, or in parallel to, Wg/Wnt signalling.

## RESULTS

### Arf6 is necessary for wing margin patterning

**Fig. 1.**
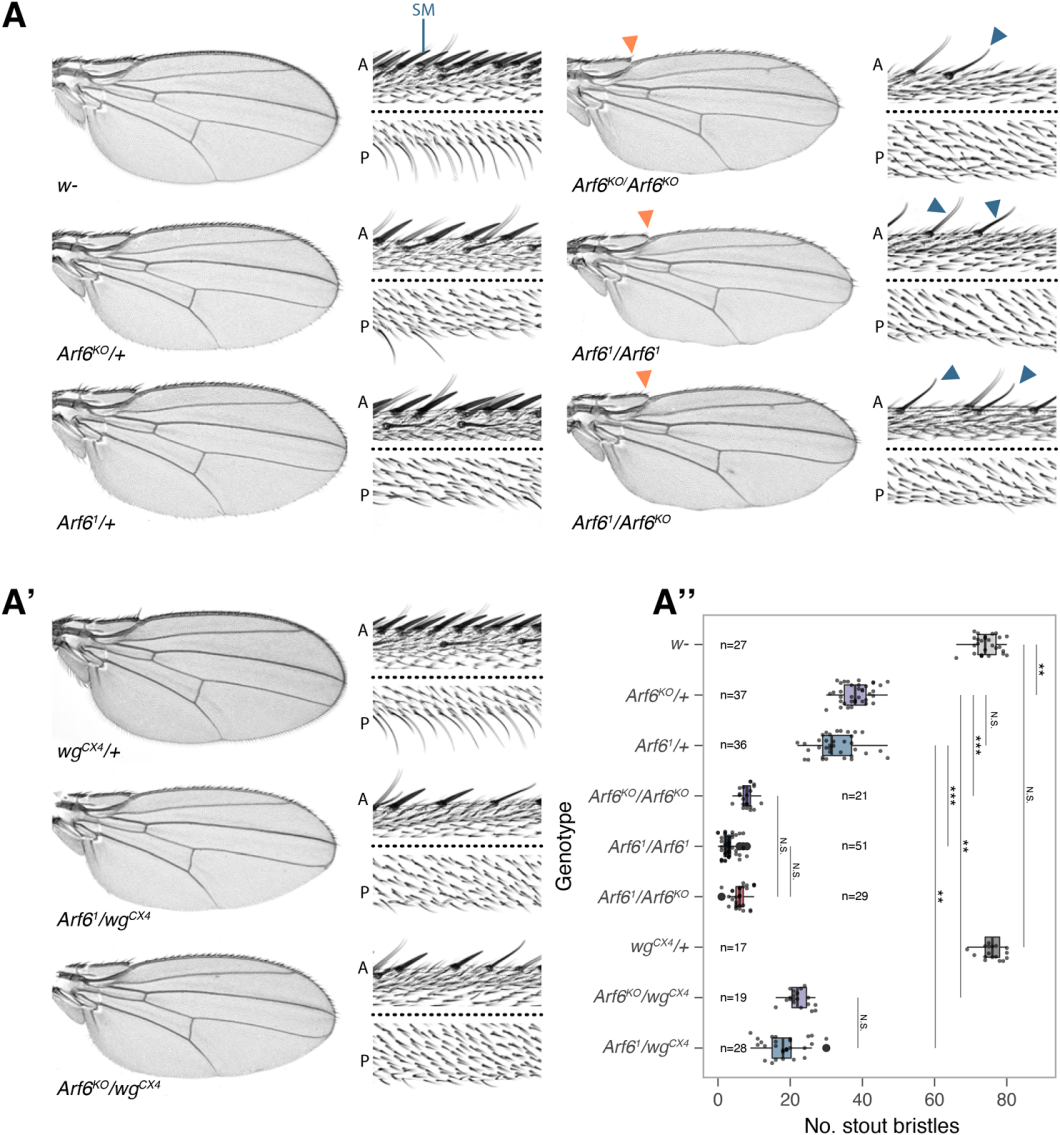
**Dominant loss of wing margin bristles in Arf6 mutants.** (A,A′) Representative wing blades and wing margins of control (*w*^−^), *Arf6^KO^*, *Arf6^1^* and *wg^CX4^* mutants and their genetic interactions. Magnifications of the anterior (A) and posterior (P) wing margins are separated by a dashed black line. Slender chemosensory bristles are still present in the homozygous *Arf6* mutants (solid blue arrowheads) while stout mechanosensory bristles (SM) are almost all absent. The solid orange arrowheads indicate the loss of distal costa bristles in *Arf6* mutants. The number of SM is quantified in A″. SM counts were analysed using a Kruskal–Wallis test. Significance values for pairwise comparisons between genotypes were calculated using a post-hoc Dunn test and reported using the following abbreviations: N.S., *P*>0.05; *, *P*≤0.05; **, *P*≤0.001; ***, *P*≤0.001.

We observed a dominant reduction in the number of bristles throughout the wing margins of adult flies heterozygous for the amorphic *Arf6* alleles, *Arf6^1^* and *Arf6^KO^* (Dyer et al., 2007; Huang et al., 2009) (Fig. 1A,A″) (see Fig. S1C for an overview of wing margin bristle patterning). This phenotype was strongly enhanced in homozygous *Arf6* mutants (Fig. 1A,A″). The trans-allelic combination of *Arf6^1^* and *Arf6^KO^* resulted in a comparable phenotype to the respective homozygotes (Fig. 1A,A″), showing that the loss of the DNA region common to both deficiencies is responsible for the phenotype (Fig. 1A; Fig. S1B).

The patterning of the wing margin is coordinated by high level Wg signalling at the dorso-ventral (D/V) boundary late in larval development (Couso et al., 1994; Jafar-Nejad et al., 2006). We therefore tested whether the *Arf6* mutant phenotype is sensitive to the level of Wg. Although the null *wg* allele, *wg^CX4^*, does not induce a dominant wing margin phenotype (Fig. 1A′,A″), when in combination with either heterozygous *Arf6^1^* or *Arf6^KO^*, it strongly enhanced the *Arf6* wing margin phenotype (Fig. 1A′,A″). We did not observe notching of the wing margin, or morphological defects in the bristles in *Arf6* mutants either alone or in combination with *wg^CX4^* (Fig. 1A,A′,A″).

### Wg-dependent senseless expression is suppressed in an Arf6 mutant

**Fig. 2.**
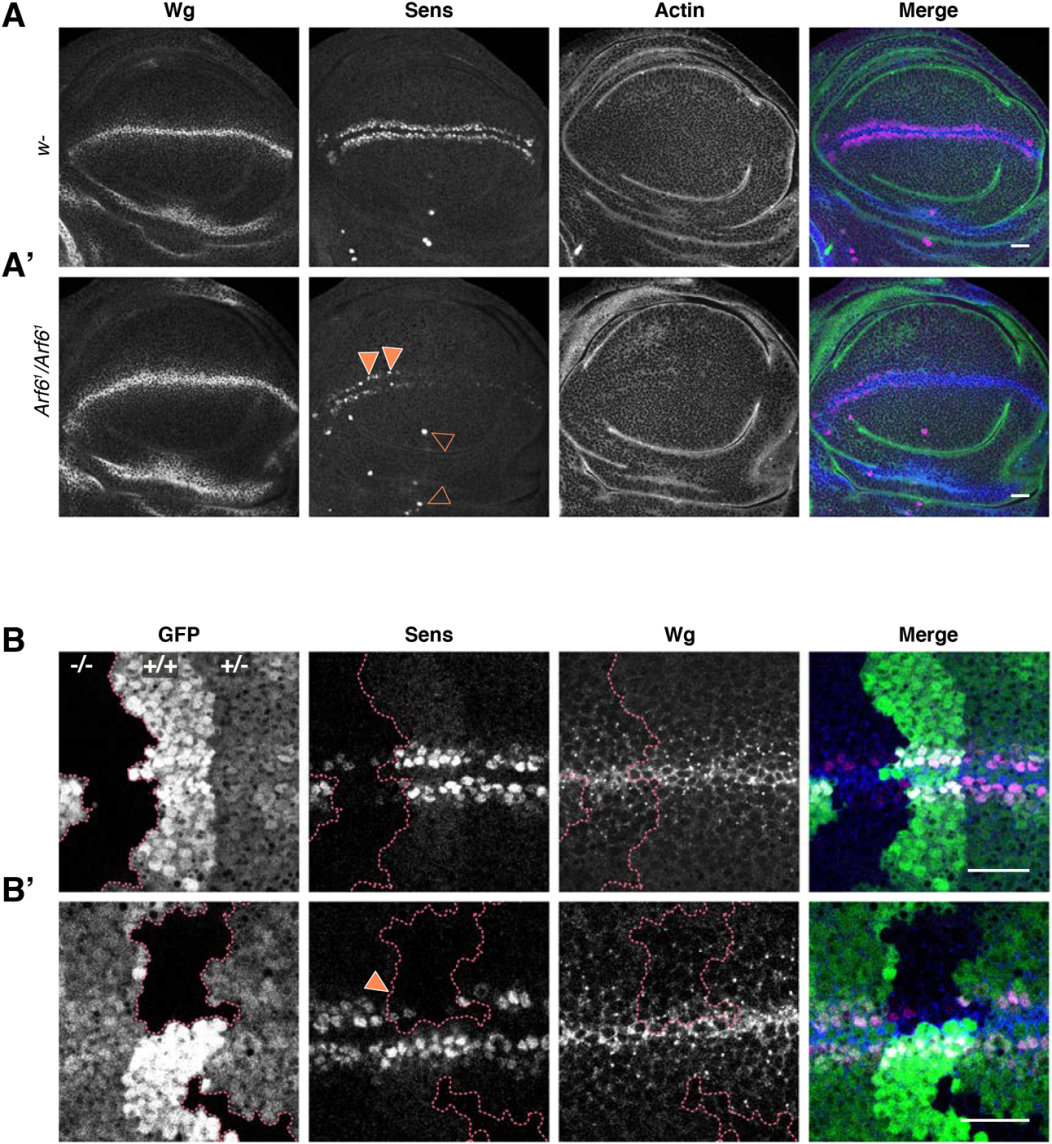
**The level of Sens expression is strongly reduced in the absence of Arf6.** (A) Wg and Sens staining in control (w^−^) and *Arf6^1^* mutant. Anterior wing margin is to the left, posterior is to the right. (A′) Sens is almost completely absent in the posterior wing margin while Sens-positive cells are occasionally observed in the anterior wing margin (closed orange arrowheads) of the homozygous *Arf6^1^* mutant. Sens is also observed in the prospective ventral radius and campaniform sensilla (open orange arrowheads). Wild-type (WT) *n*=10, *Arf6^1^ n*=10. (B) Sens and Wg staining in *Arf6^1^* mutant clones is marked by the absence of GFP (^−^/^−^). Heterozygous and homozygous wild-type tissues are marked by (+/−) and (+/+), respectively. In the merged images, Sens is in magenta, Wg in blue, GFP in green (B,B′) and actin in green (A,A′). *n*=18 (B′) a strong reduction in Sens staining is observed in clones that do not enter the Wg expression domain. Scale bars: 20 µm. *n*=19.

The zinc finger transcription factor Sens acts as the proneural factor for many of the margin bristles and is expressed in two narrow stripes flanking the D/V boundary in response to high level Wg signalling (Jafar-Nejad et al., 2006; Nolo et al., 2000) (Fig. 2A). Sens staining was strongly reduced throughout the presumptive wing margin in an *Arf6* mutant wing disc, but not in the sensory organ precursor in which the expression of Sens is independent of Wg (Fig. 2A′). The bristles induced by ectopically expressing *sens* were not dominantly suppressed in the *Arf6* mutant, indicating that the loss of bristles was not due to a loss of Sens proneural activity (Fig. S2A,A′).

To test whether the reduction in Sens is due to a defect in *wg* expression, we analysed the pattern of Wg in *Arf6^1^* mutant wing discs (Fig. 2A). The Wg stripe at the D/V boundary was not disrupted by the loss of *Arf6*. Interestingly, the low-threshold Wg target Distal-less (Dll) was not reduced in *Arf6* mutant conditions (Fig. S3A,A′,B) indicating that Arf6 is not necessary for low level Wg signalling.

In order to assess whether Arf6 is required cell autonomously in Wg signal transduction, we generated random mitotic *Arf6^1^* clones that we then stained for Sens and Wg. Consistent with the dominant loss of bristles in *Arf6* mutants, we observed a strong reduction in Sens staining in homozygous *Arf6^1^* clones, an intermediate level in heterozygous tissue and the wild-type levels in the wild-type tissue (Fig. 2B,B′). Importantly, clones that overlapped with the *sens* expression domain, without entering the *wg* expressing margin cells, still induced a strong reduction in Sens staining (closed orange arrowheads, Fig. 2B′), demonstrating that removing Arf6 activity cell autonomously suppresses Sens in Wg receiving cells. Importantly, we did not observe ectopic Wg expression in *Arf6* clones near the D/V boundary (Fig. 2B,B′), nor wing notching in the adult *Arf6* mutant wing (Fig. 1), indicating that the integrity of the D/V boundary was not affected by the loss of *Arf6* (Rulifson and Blair, 1995; Rulifson et al., 1996). Altogether, these data show that while Arf6 is not required for the integrity of the D/V boundary, its activity is required cell autonomously for the transduction of high level Wg signalling controlling the expression of *sens* necessary for wing margin bristle development*.*

### Arf6 is necessary downstream of armadillo stabilisation

**Fig. 3.**
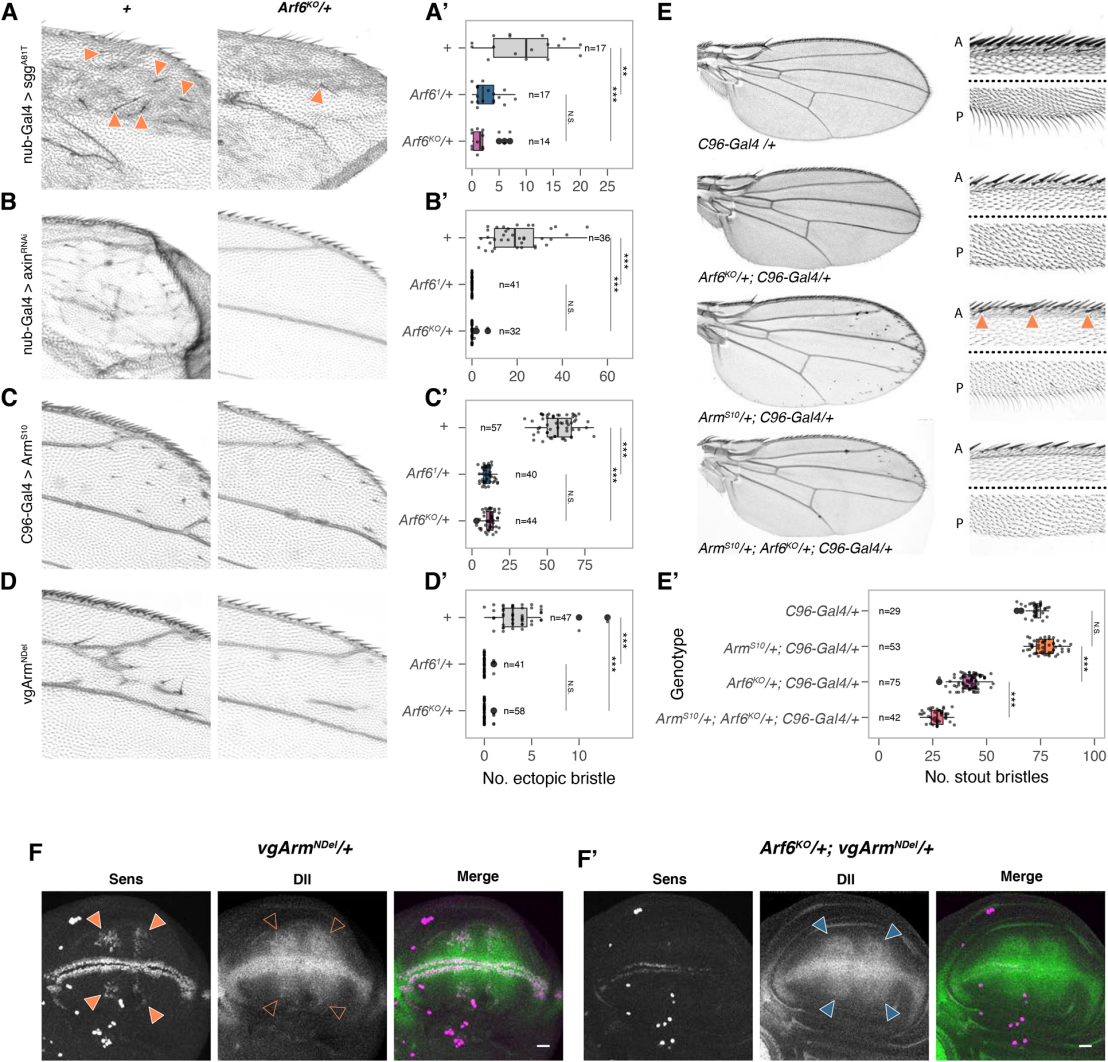
**Epistatic analysis shows that Arf6 acts downstream of Arm stabilisation.** (A) Dominant negative Sgg (*sgg^A81T^*) overexpressed with *nub-Gal4* induces ectopic bristles (closed orange arrowheads), which are dominantly suppressed in the *Arf6* mutant background (quantification in A′). (B) Knock-down of *axin* induces ectopic bristles (B′), which are dominantly suppressed in the *Arf6* mutant background. (C) *Arm^S10^* (expressed with C96-Gal4) and (D) *vgArm^NDel^* (expressed under *vestigial* margin and quadrant enhancers) introduce ectopic bristles that are dominantly suppressed in the *Arf6* mutant background (quantified in C′ and D′). (E) *Arm^S10^* expression with *C96-Gal4* at 25°C enhances *Arf6^KO^* margin phenotype, but introduces ectopic margin bristles in a wild-type background (solid orange arrowheads). (E′) Quantification of stout mechanosensory bristles. Bristle counts were analysed using a Kruskal–Wallis test. Significance values for pairwise comparisons between genotypes were calculated using a post-hoc Dunn test and reported using the following abbreviations: N.S., *P*>0.05; *, *P*≤0.05; **, *P*≤0.001; ***, *P*≤0.001. (F) vgArm^NDel^ expression induces ectopic Sens (closed orange arrowheads) and Dll (open orange arrowheads) in a wild-type background. (F′) Ectopic Sens, but not Dll (closed blue arrowheads) is suppressed in a heterozygous *Arf6^KO^* background. In the merged images, Sens is in magenta, Dll in green.

In order to determine the level at which Arf6 is required in Wg signal transduction, we began by activating the Wg signalling pathway in an *Arf6* mutant background. We suppressed the activity of the destruction complex by expressing a dominant-negative form of the *Drosophila* GSK3β homologue, *shaggy* (*sgg^A81T^*) (Bourouis, 2002) or knocking-down *axin*. Both treatments induce high level Wg signalling and the formation of ectopic bristles in the wing blade (Fig. 3A,B). The number of ectopic bristles was dominantly suppressed in heterozygous *Arf6* mutant backgrounds (Fig. 3A,A′,B,B′). These data indicate that the loss of bristles and Sens expression in the *Arf6* mutants is not a result of the hyperactivation of the Arm destruction complex, and suggest that Arf6 acts downstream of Arm stabilisation.

We next confirmed that Arf6 acts downstream of the stabilisation of Arm by expressing two constitutively active forms of Arm: Arm^S10^ and Arm^NDel^ (Pai et al., 1997). Importantly, these N-terminally truncated forms of Arm accumulate in the cytoplasm, triggering constitutive, high level Wg signalling in a ligand independent manner (Pai et al., 1997; Somorjai and Martinez-Arias, 2008). We expressed Arm^S10^ in a broad domain overlapping the D/V boundary with the *C96-Gal4* driver, while Arm^NDel^ expression is directly driven by the *vestigial* quadrant and margin enhancers (subsequently referred to as *vgArm^NDel^*). Both Arm variants induced ectopic bristles in the wing blade (Fig. 3C,C′,D,D′). Importantly, the bristles induced by vgArm^NDel^ were not dependent on endogenous Wg signalling (Fig. S4A,A′,B,B′,B″) and vgArm^NDel^ is active in canonical Wg signalling (Fig. S4C). The ectopic bristles induced by both constructs were dominantly suppressed in the *Arf6* mutant background (Fig. 3C,C′,D,D′). Moreover, vgArm^NDel^ or Arm^S10^ did not rescue the wing margin bristles lost in the wing margin of *Arf6^KO^* flies, and instead caused an enhancement of the *Arf6* mutant phenotype (Fig. 3E,E′; Fig. S5A,A′). Over-expressing wild-type *dsh* also induced ectopic bristles that were suppressed in a heterozygous *Arf6^KO^* background (closed orange arrowhead, Fig. S5B,B′). *dsh* over-expression also enhanced of the heterozygous *Arf6^KO^* phenotype **(**compare Fig. S5B,C,C′). This is unlikely to be due to a dominant negative effect of Arm^S10^ or Dsh overexpression as expressing either of these constructs in a wild-type background did not induce wing margin defects. Moreover, we did not observe a change in the levels of endogenous Arm and Cadherin at the adherens junctions in *Arf6^1^* mutant clones (Fig. S6A,A′), suggesting that Arf6 does not regulate Wg signalling through the sequestration of Arm to the adherens junction in *Drosophila* (Grossmann et al., 2013; Pellon-Cardenas et al., 2013). Altogether, these data demonstrate that Arf6 is required genetically downstream of Arm stabilisation in order to activate high level Wg signalling.

To test whether stabilised Arm had a generally reduced signalling activity in the *Arf6* mutants, we stained for both Sens and Dll in wing imaginal discs expressing *vgArm^NDel^* in either a wild-type (Fig. 3F) or heterozygous *Arf6^KO^* background (Fig. 3F′). Clusters of ectopic Sens positive nuclei were apparent far from the D/V boundary in control wing discs expressing *vgArm^NDel^* (closed orange arrowheads, Fig. 3F) accompanied by an upregulation of Dll (open orange arrowheads, Fig. 3F). Removing a single copy of *Arf6* led to an almost complete suppression of the ectopic Sens expression, including at the D/V boundary but both the ectopic and endogenous Dll remained (closed blue arrowheads, Fig. 3F′). These data indicate that although vgArm^NDel^ is still able to activate low level signalling in the *Arf6* mutant background, its ability to activate Sens expression is strongly attenuated. Importantly, although the *Arf6* margin phenotype was mildly enhanced in a heterozygous *arf1 (arf1^182-1^)* mutant background, the signalling activity of Arm^NDel^ was not suppressed in a heterozygous *arf1^182-1^* background (Fig. S7A,A′,B,B′). This suggests that although Arf1 contributes to wing patterning, it likely does so in a distinct manner to Arf6 (Hemalatha et al., 2016).

Together, these results emphasise the specific requirement for Arf6 for the cell autonomous establishment of *sens* expression in response to high level Wg signalling. The loss of margin bristles in the *Arf6* mutants is therefore likely to be due to a loss of the Sens-positive proneuronal clusters of the wing margin due to a suppression of high level Wg signalling.

### Arf6 is necessary at the level or upstream of Pangolin

The dominant suppression of N-terminally truncated Arm activity in *Arf6* mutants suggests that Arf6 could be involved in positively regulating canonical nuclear Wg signalling. Pavarotti (Pav), a MKLP1 homologue (Dyer et al., 2007; Makyio et al., 2012) has previously been shown to act in the nucleus as a negative regulator of Wg signalling during embryonic development (Jones et al., 2010). MKLP1 also recruits, and physically interact with Arf6 at the flemming body during cytokinesis (Makyio et al., 2012). We therefore hypothesised that Pav could provide the functional link between Arf6 and Wg signalling.

**Fig. 4.**
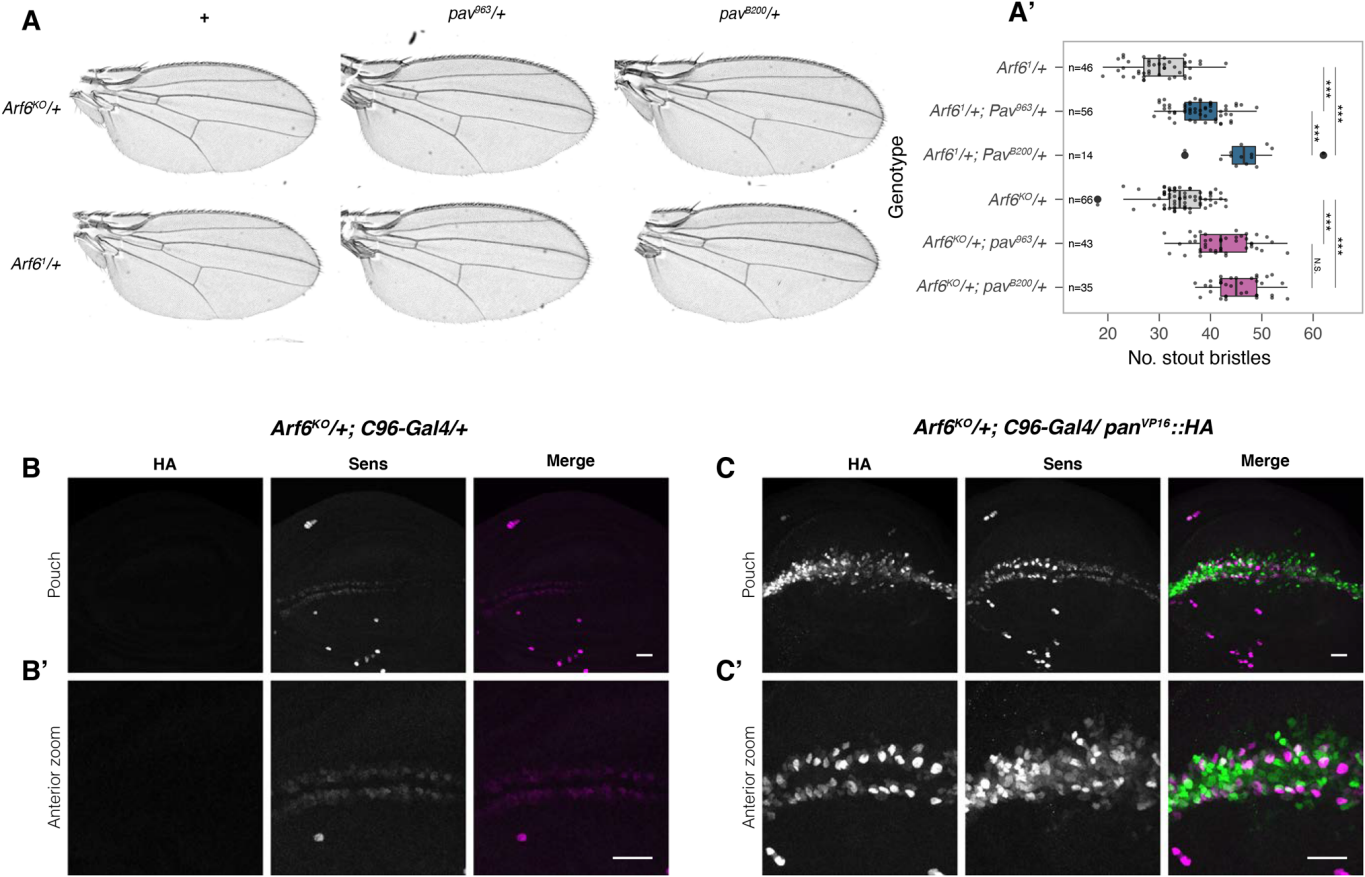
**Removal of one copy of pav, as well as Pan-VP16::HA overexpression rescue the Arf6 phenotype.** (A) The *Arf6* mutant phenotype is partially rescued in a hypomorphic *pav* background (stout mechanosensory bristles quantified in A′). (B) Wing imaginal discs showing Sens expression in *Arf6^KO^/+* and in (C) *Arf6^KO^/+* with Pan-VP16::HA expressed with *C96-Gal4*. Anterior magnification of control and rescue discs are presented in B′ and C′ (in the merged images, HA is magenta and Sens is green).

We began by testing whether the *Arf6* phenotype is sensitive to changes in the level of Pav. Pav is essential during cytokinesis (Adams et al., 1998), we therefore opted to use hypomorphic *pav* alleles (*pav^B200^* and *pav^963^*) to avoid strong pleiotropic effects. Heterozygous *pav^B200^* and *pav^963^* flies in a heterozygous *Arf6* background provided a partial rescue of the number of wing margin bristles (Fig. 4A,A′) in the wing margin. These conditions did not induce cytokinesis defects or wing notching (Fig. 4A; Fig. S8), consistent with Arf6 being dispensable for somatic cytokinesis in *Drosophila* (Dyer et al., 2007). The genetic interaction between *Arf6* and *pav* indicate that Arf6 could be regulating nuclear Wg signalling by modulating the non-canonical activity of Pav as a negative regulator of Pan activity (Jones et al., 2010).

Once in the nucleus, Arm forms a complex with Pan, a TCF/LEF homologue forming the core of the enhanceosome (Gammons and Bienz, 2018). To determine whether Arf6 acts upstream of the enhanceosome, we generated a constitutively active form of Pan (Pan-VP16::HA, see Materials and Methods) (Fig. S9A,SA′). Expressing *pan-VP16::HA* in a wild-type background only induced low levels of ectopic Sens expression (Fig. S9B; closed orange arrowheads, Fig. S9B′), and was not sufficient to activate *sens* expression far from the D/V boundary (open orange arrowheads, Fig. S9B′), indicating that its activity still requires endogenous permissive signals. Expressing *Arm^S10^* under the same conditions induced extensive ectopic Sens throughout the C96 expression domain (Fig. S9C,C′). Despite its greater ability to induce Sens expression, expressing *Arm^S10^* with *C96-Gal4* in a heterozygous *Arf6^KO^* background did not rescue Sens expression (Fig. S9D,D′), whilst expressing *pan-VP16::HA* in the same conditions resulted in a substantial rescue of Sens throughout the D/V boundary (Fig. 4C,C′). Taken together, these results indicate that Arf6 activity is required genetically downstream of the stabilisation of Arm, but upstream or at the level of Pan activity for the induction of *sens* expression.

## DISCUSSION

We have demonstrated a novel requirement for the small GTP binding protein Arf6 during *Drosophila* wing development. The *Arf6* mutant phenotype is characterised by a dominant reduction in the number of bristles in the adult wing margin, accompanied by reduced *sens* expression in the wing margin PNCs in the wing imaginal discs. The patterning of the wing margin requires the expression and activity of Sens in the cells flanking D/V boundary in response to high level Wg signalling activity (Jafar-Nejad et al., 2006; Nolo et al., 2000). *sens* begins to be expressed in this compartment late in larval development and reducing Wg signalling during this period is associated with similar phenotypes to those we observed in the *Arf6* mutant background (Couso et al., 1994). We therefore focused on understanding the *Arf6* mutant phenotype in the context of Wg signalling. Based on epistatic interactions, we established that Arf6 acts genetically downstream of the stabilisation of Arm, but upstream or at the level of nuclear Pan activity for the expression of *sens* in response to Wg signalling. As Arf6 acts at the plasma membrane and endosomal membranes, it is unlikely to directly regulate nuclear Wg signalling (Donaldson and Jackson, 2011). We therefore suggest that Arf6 could regulate Wg signalling through the non-canonical activity of the MKLP1 orthologue, Pav, previously shown to directly interact with Arf6, and to act as a nuclear repressor of Wg signalling during *Drosophila* embryogenesis (Jones et al., 2010). This could be achieved through the sequestration of Pav to endosomal membranes by Arf6, preventing its access to the nucleus.

Our findings complement the results of previous *in vitro* studies in which Arf6 was shown to act upstream in Wnt signalling at the level of signalosome activity, or through reallocation of junctional β-catenin to the cytoplasm (Grossmann et al., 2013; Kim et al., 2013; Pellon-Cardenas et al., 2013). These findings are not mutually exclusive, as it is not yet clear whether the downstream role of Arf6 is conserved in Wnt signalling, as Wnt conditioned medium was used as a source of Wnts, meaning that a role for Arf6 in upstream signalling steps would likely mask a potential downstream role. A downstream role of Arf6 in Wnt signalling would be of particular relevance to pathologies such as colorectal and breast cancers induced by hyperactivation of Wnt signalling (Zhan et al., 2017). This is most commonly a result of mutations in components of the β-catenin destruction complex, or more occasionally β-catenin itself, leading to β-catenin stabilisation (Clevers and Nusse, 2012). Wnt signalling in these contexts is ligand-independent, making downstream regulators of Wnt transduction potentially valuable therapeutic targets. Small molecule inhibitors of Arf6 have already been identified, and Arf6 inhibition in adults has not been associated with secondary effects (Grossmann et al., 2019; Macia et al., 2021).

The *Drosophila Arf6* phenotype is particularly striking due to it being dominant, while specifically impacting a high threshold Wg signalling target, *sens*, without affecting the low threshold target *dll*. These observations can be interpreted as Arf6 specifically acting in the transduction of high threshold Wg signalling, as *sens* has previously been shown to be much more sensitive to perturbations in Wg signalling than other Wg targets such as *dll* or *vestigial* (*vg*) (Baena-Lopez et al., 2009; Song et al., 2009). However, we cannot exclude the possibility that Arf6 is required for a process acting in parallel to Wg signalling, specifically necessary for the induction of *sens* expression in response to high level Wg signalling. Although *sens* expression is frequently used as a readout of Wg signalling, little is known about the regulatory logic and temporal dynamics underlying its regulation by Wg signalling*.* Furthermore, the wing margin PNCs represent one of the few known contexts in which *sens* expression is regulated by Wg signalling rather than by the bHLH proneural proteins, Achaete (Ac) and Scute (Sc) (Jafar-Nejad et al., 2006; Nolo et al., 2000; Vincent, 2014). In contrast to *Arf6* mutants, flies lacking both *ac* and *sc* lose the majority of sensory organs throughout the body, while the stout mechanosensory organs, and non-innervated bristles of the wing margin remain (García-Bellido and De Celis, 2009; Jack et al., 1991; Jafar-Nejad et al., 2006). This, combined with lack of a more general defect in bristle development in the *Arf6* mutant indicates that the *Arf6* mutant affects the Wg-dependent regulation of *sens.* This is particularly pertinent in the posterior compartment of the wing disc, in which the bHLH proneural factors are not expressed*.* Understanding the mechanism underlying the *Arf6* mutant phenotype could provide insights into the cellular response to different levels of Wg signal transduction, and into the regulation of *sens* expression during wing margin development*.*

The high level of conservation of Arf6 and the Wg signalling pathway makes the molecular mechanism underlying the *Arf6* phenotype more likely to be relevant beyond *Drosophila* wing development. Identifying the Arf6 regulators and effectors relevant to wing margin development, and in turn whether Arf6 activity is regulated by Wg signalling will not only help to understand the *Arf6* phenotype, but could also provide more general insights into the mechanisms governing Arf6 activity in patho-physiological conditions.

## MATERIALS AND METHODS

### Fly genetics

Flies were raised in standard conditions. Crosses were carried out at 22°C unless stated otherwise.

### Clone induction

Clones were generated by crossing males of either *FRT42B*, *Arf6^KO^/CyO*, *Tb::RFP* or *FRT42B*, *Arf6^1^/CyO*, *Tb::RFP* with virgins of *y^1^*, *w^1118^*, *hsFLP; FRT42B*, *ubi-nlsGFP*. Heat shock induction was carried out for 30 min in a water bath at 37°C, 48 h after egg lay. Larvae carrying *Arf6^1^* or *Arf6^KO^* were selected based on the absence of Tb, then dissected and stained in wandering stage L3. Mutant clones were recognised based on the absence of a GFP signal.

### Fly stocks

The following fly stocks were used during this study: *w^1118^* (Bloomington #3605) served as a wild-type control and the source of wild-type chromosomes. *Arf51F^GX16w^*^−^ (*Arf6^KO^*) (Bloomington #60585; Huang et al., 2009), *Arf6^1^* (Dyer et al., 2007) (a kind gift from Marcos Gonzalez Gaitan, Université de Genève) are both independently generated null alleles of *Arf6* lacking the full coding region. *Arf6^KO^* was initially recessive lethal, so we introgressed both *Arf6* null alleles into a *w-* background for five generations and reconfirmed the presence of the deletions by PCR. *Arf6^KO^* and *Arf6^1^* were maintained as a stock balanced over *CyO, Tb::RFP* (Bloomington #36336) to allow homozygous larvae to be recognised. *arf79F^182-1^* is a null allele of *Drosophila arf1* (referred to as *arf1^182-1^* in text) and was a kind gift from Tony Harris, University of Toronto. *ARF6::GFP* (Bloomington #60586) is an endogenous, C-terminally tagged form of Arf6 generated in the *Arf6^KO^* background (Huang et al., 2009). High level Wg activation was induced using *UAS-dsh::myc* (Bloomington #9453), *UAS-sgg^A81T^* (Bloomington #5360) (Bourouis, 2002), *UAS-Arm^S10^* (encoding Arm lacking amino acids 37 to 84 in the N-terminus, Bloomington #4782) (Pai et al., 1997), *vgMQ-arm^NDel^* (expresses a form of Arm lacking amino acids 1 to 138 from the N terminus, Bloomington #8370) or *UAS-axin-RNAi* (Bloomington #31705). Wg signalling was induced downstream of Arm stabilisation was achieved using *UAS-pan^VP16^::HA* (generated in this study, see methods below).

Wg signalling suppression was achieved with *UAS-dsh-RNAi* (KK330205, VDRC), *UAS-arr-RNAi* (GD6707 and GD6708, VDRC) or *wg^CX4^* (Bloomington #2980). Wild-type *sens* was over-expressed with *UAS-sens* (Bloomington #42209). The following Gal4 drivers were used to drive expression in the wing imaginal disc: *nubbin-Gal4* (expressed throughout the wing pouch) (Azpiazu and Morata, 2000) *C96-Gal4* (expressed in a wide domain overlapping the D/V boundary) (Bloomington #43343). Mitotic clones were induced *using y,w,hsFLP*; *FRT42B*, *ubi-GFP^NLS^* (derived from Bloomington #5826), and *Arf6^KO^*, *FRT42B/CyO*, *Tb::RFP* or *Arf6^1^*, *FRT42B/ CyO*, *Tb::RFP* (derived from Bloomington stocks #1956 and #36336).

The following independently generated EMS-induced *pav* alleles were used: *pav^B200^* (Bloomington #4384) (Salzberg et al., 1994) and *pav^963^* (Bloomington #23926) (Collins and Cohen, 2005).

### Generating pan^VP16^::HA

*pan^VP16^::HA* was generated in order to allow the induction of Wg signalling downstream of Arm stabilisation. The construct is conceptually based on a construct previously shown to act independently of enhanceosome components Legless (Lgs) and Pygopus (Pygo) (Thompson, 2004). A sequence encoding full length Pan, excluding the stop codon, followed by 3xHA flanked by GGGGS linkers, and finally the *VP16* transcriptional activation domain was synthesised (GeneArt). The sequence was directionally subcloned into 5′ KpnI and 3′ XbaI into *pUAST attb L34* plasmid (Bischof et al., 2007). Purified maxipreps were injected into the *M{3xP3-RFP.attP'}ZH-68E* background (Bl# 24485) (Bischof et al., 2007) in order to generate third chromosome insertions.

### Antibodies

The following primary antibodies were used: rabbit anti-GFP (1:400, Life Technologies A6455), Guinea pig anti-Sens (1:1000, a kind gift from Hugo Bellen, Baylor College of Medicine), rat anti-Distalless (1:100, a kind gift from Marc Bourouis, Institut de Biologie Valrose), mouse Anti-Wg (1:100, DSHB 4D4), mouse anti-Arm (1:10 DSHB N2 7A1). Rat anti-DE-cadherin (1:50, DSHB DCAD2).

The following secondary antibodies were used: goat anti-rabbit Alexa488 (1:500; Invitrogen A11034), goat anti-rabbit Alexa546 (1:500; Invitrogen A11035), donkey anti-mouse Alexa488 (1:500; InvitrogenA21202), donkey anti-mouse Alexa546 (1:500; Invitrogen A10036), donkey anti-rat Alexa488 (Invitrogen A21208), goat anti-rat Alexa546 (1:500; Invitrogen A11081) and TRITC-phalloidin (1:100; Sigma-Aldrich P1951-1MG).

### Wing imaginal disc preparation and imaging

Wandering stage L3 larvae were washed then dissected in ice-cold 1xPBS. Fixation was carried out for 20 min at room temperature in 3.7% formaldehyde with constant agitation. Samples were washed and permeabilised for 30 min in PBT (0.3% Triton X-100, 1x PBS) then blocked for 1 h in blocking buffer (0.1% Triton X-100, 1% BSA, 1x PBS) at room temperature. Primary antibody incubations were carried out overnight at 4°C in 200 µl of antibody diluted in blocking buffer. Samples were washed 3x 20 min in PBT, then incubated for 1 h at room temperature with secondary antibodies. Samples were washed in PBT then mounted in VECTASHIELD mounting medium (Vector Laboratories).

Images were acquired with a Leica TCS upright SP5 confocal microscope using a 40x objective (HCX PLAN APO; Numerical aperture of 1.3). The Leica LAS AF software package was used for image capture (v 2.6.3.8173). Images were analysed using FIJI (Schindelin et al., 2012) and the data analysed and visualised in R (R Core Team, 2020). Data-points were overlayed on the boxplots to display data distribution. Larger points represent numerical outliers, defined as points that fall outside 1.5x the interquartile range, above the upper, and below the lower quartiles.

### PCR validation of Arf6 deficiencies

Genomic DNA was extracted from individual flies. Flies were crushed in PCR tubes using a pipette tip containing 50 μl of squashing buffer (10 mM Tris-HCl, 1 mM EDTA, 25 mM NaCl and 200 μg/ml proteinase K). Samples were incubated at 37°C for 30 min then heat inactivated at 95°C for 2 min using a thermocycler. 1 μl of the resulting extraction was used as the PCR template.

The deficiency described for *Arf6^1^* was validated using PCR (Fig. S1B′) and the primer combinations shown in (Fig. S1B). *Arf6^KO^* has previously been characterised in Huang et al. (2009). Primer sequences used are provided in the table below. 2x GoTaq Green Master Mix (M7121, Promega) was used for the PCR reactions. The following primers were used to validate the *Arf6^1^* allele:

**Table:**
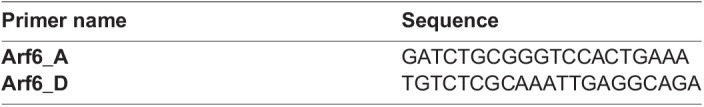


### Adult wing dissection

Adult flies were collected in ethanol at least 12 h following emergence to ensure their wings had fully expanded and dried. Wings were removed at the hinge in ethanol, dried on blotting paper, then mounted in a drop of Euparal (Carl Roth #7356.1) and left to cure overnight on a slide heating plate set at 60°C. Wings were imaged using a Leica DM2000 with an attached Leica DFC7000T camera. Wings were excluded from quantifications if damage to the wing margin prevented bristle quantification.

### Quantification and statistical analysis

The numbers of both ectopic and stout wing margin bristles (Fig. S1C) were quantified manually using the cell counter plugin in FIJI (Schindelin et al., 2012). Statistical analyses and plotting were carried out in R (version 3.6.3) (R Core Team, 2020). The counts of both stout bristles and ectopic bristles for multiple genotypes were analysed using the Kruskal–Wallis test. Post-hoc pairwise comparisons between the counts for individual genotypes were carried out using the Dunn test. The *P*-values resulting from multiple comparisons were corrected for Type 1 error using the Benjamini–Hochberg procedure. Single comparisons were made using Mann–Whitney U tests. Plots were generated using the GGPLOT2 package and exported using the egg package (Auguie, 2019; Wickham, 2009). Sample sizes are marked on the plots or provided in figure legends.

## Supplementary Material

Supplementary information
