# Supplementary material for: Arf6 is necessary for senseless expression in response to wingless signalling during Drosophila wing development
Source: Biol Open. 2021 Dec 2;10(12):bio058892. doi: 10.1242/bio.058892 (PMC8656404; doi:10.1242/bio.058892)
Supplement: Supplementary information [file biolopen-10-058892-s1.pdf]

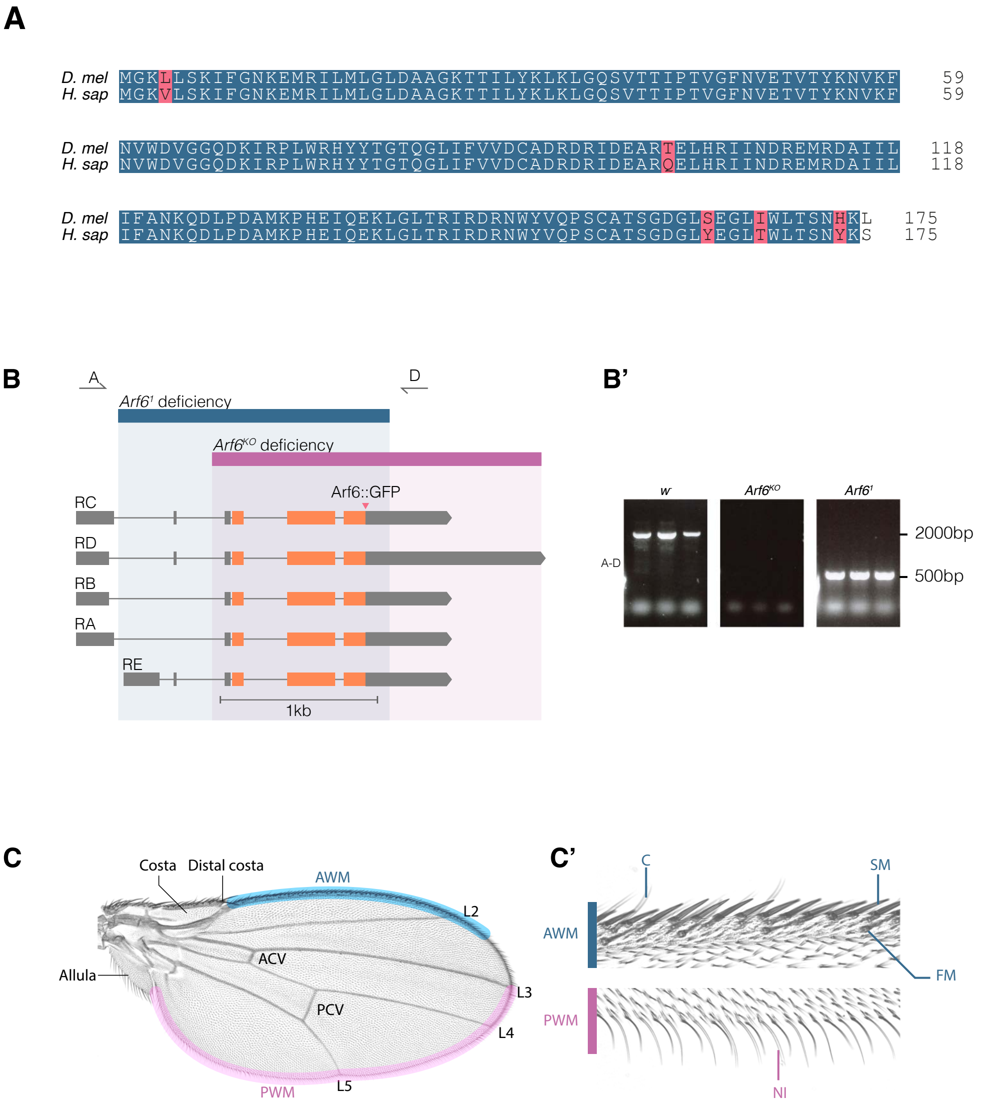

Biology Open • Supplementary information

Fig. S1. The deficiencies of the Arf6^1 and Arf6^{KO} alleles delete the complete Arf6 coding region.

(A) An alignment of the primary protein sequences of Drosophila and human Arf6. There is a 97% identity conservation between the two proteins. Non-conserved residues are highlighted in pink. **(B)** the break points and deficiencies of the two null *Arf6* alleles used in this study (*Arf6*^{1} and *Arf6*^{KO}). Both deficiencies delete the complete *Arf6* coding region (shown in orange) of all predicted *Arf6* isoforms (RC, RD, RB, RA and RE). Primers are represented by half arrows above (see Table S1 for primer sequences). Thin horizontal grey lines represent intronic regions, while the grey and orange blocks represent exons. **(B')** the PCR products resulting from the PCR testing the location of the *Arf6*^{1} deletions. A 2000bp amplicon is present in control (*w*$^-$) samples whereas no signal in present in *Arf6*^{KO} due to loss of the region to which primer D binds. A 500bp band is present in *Arf6*^{1} due to amplification of regions flanking *Arf6*^{1} deletion. Three biological replicates are shown for each genotype. The deficiency in *Arf6*^{KO} was analysed in details in (Huang et al., 2009) (**C)** An outline of the main morphological features of the adult wing**:** AWM = anterior wing margin, PWM = posterior wing margin, ACV = Anterior crossvein, PCV = posterior crossvein. Details of the AWM and PWM in **(C')**. C = chemosensory bristle, SM = stout mechanosensory bristle, FM = fine mechanosensory bristle, NI = non-innervated bristle. The stout mechanosensory and non-innervated bristles are collectively referred to as the margin bristles in the text.

Biology Open • Supplementary information

Biology Open (2021): doi:10.1242/bio.058892: Supplementary information

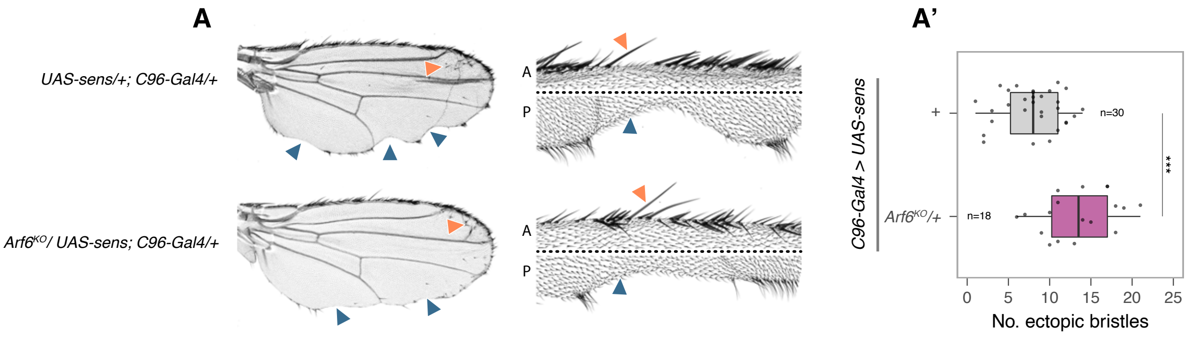

Fig. S2. Sens proneural activity is not impaired in an Arf6 mutant

(A) Over-expressing wild-type Sens induces ectopic bristles (closed orange arrowheads) and wing margin notching (closed blue arrowheads) in both a wild-type and *Arf6*^{KO} background, resembling the previously described hypermorphic *sens*^{Lyra} allele (Nolo et al., 2001). **(A')** the number of ectopic bristles induced by Sens over-expression is not reduced in an Arf6 mutant background, indicating that the proneural capacity of Sens was not suppressed in a heterozygous *Arf6* mutant background. Ectopic bristle numbers were analysed using a Kruskal-Wallis test. Significance values for pairwise comparisons between genotypes were calculated using a post-hoc Dunn test and reported using the following abbreviations: N.S. = $p > 0.05$, * = $p \leq 0.05$, ** = $p \leq 0.001$, *** = $p \leq 0.001$.

Biology Open • Supplementary information

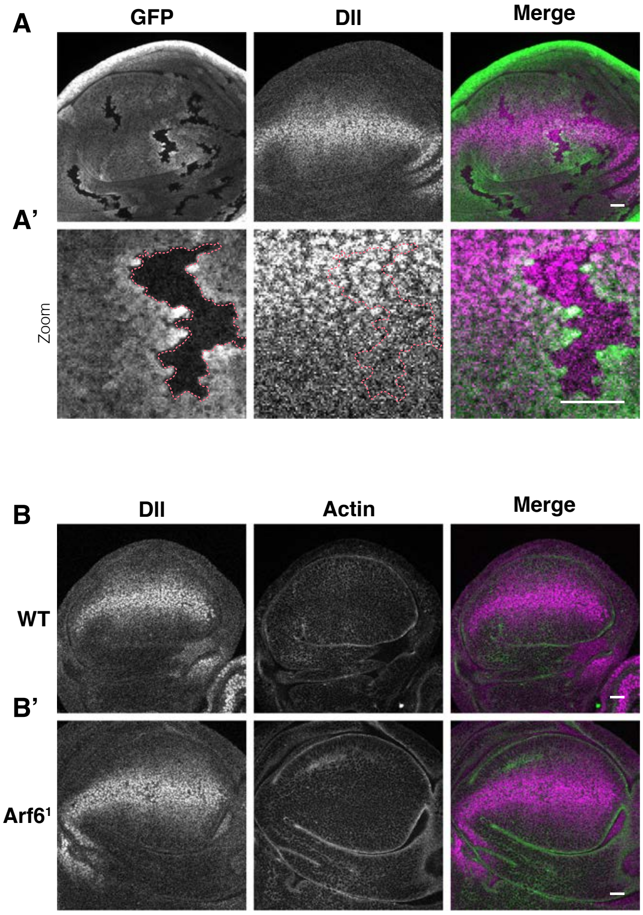

Fig. S3. distalless expression is not affected in the absence of Arf6.

(A) Dll staining in *Arf6*^{1} clones (labelled by the absence of GFP) with zoom shown in **(A')** n=23**. (B)** Dll staining in both WT and homozygous Arf6^{1} mutant discs **(B')**. In the merges, Dll is in magenta, GFP in green (B-B') and actin in green (B''). All scale bars represent 20µm. WT n=5, Arf61 n = 8

Biology Open • Supplementary information

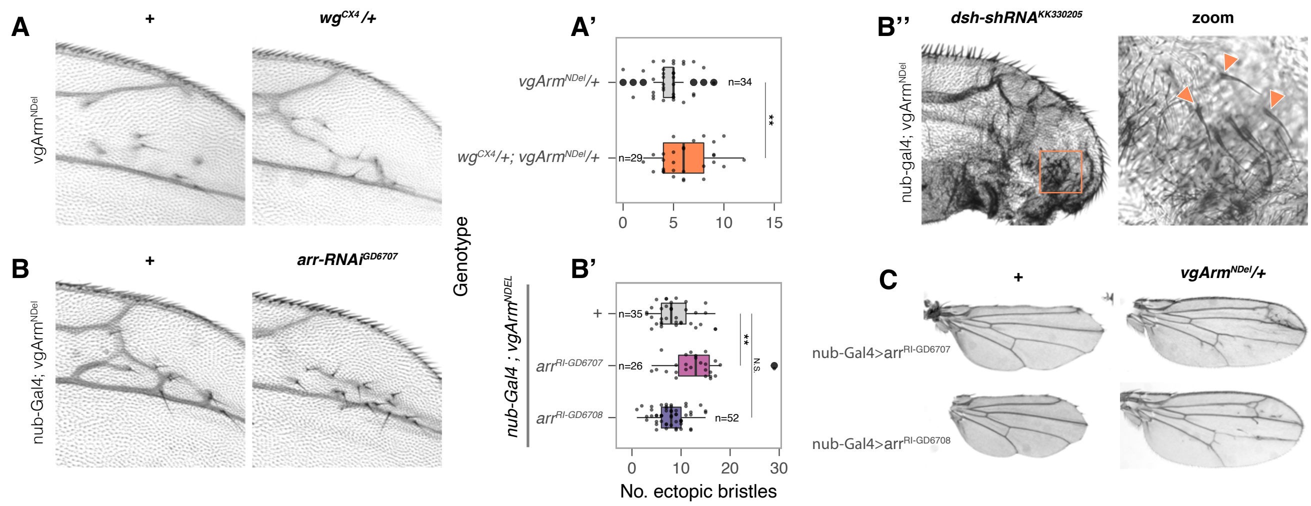

Fig. S4. vg$_{MQ}$-Arm^{NDel} acts independently of endogenous Wg signalling

(A) The ectopic bristles induced by vg$_{MQ}$-Arm^{NDel} (vgArm^{NDel}) are not dominantly reduced in a heterozygous *wg* background (*wg^{CX4}/+*) indicating that vgArm^{NDel} activity is independent of endogenous Wg ligand and signalosome activity. (**A**') The quantification of the number of ectopic bristles. (**B)** knocking down the Wg co-receptor *arr* with *nub-gal4* also does not suppress the formation of ectopic bristles induced by vgArm^{NDEL}, again suggesting that the signalling activity of vgArm^{NDel} does not depend on endogenous Wg signalling **(**quantified in **B')**. (**B'') Knock-down of dsh caused strong wing defects, but does not suppress the activity of vgArm^{NDEL}. No quantification is shown due to the difficulty of reliably discerning ectopic bristles in this context. **(C)** vgArm^{NDEL} is able to rescue the loss of the wing margin loss induced by knocking down *arr* with nub-gal4. This shows that vgArm^{NDel} can recapitulate WT canonical wingless signalling activity and is sufficient to rescue a moderate Wg wing margin phenotype induced by the *arr* knock-down.

Biology Open • Supplementary information

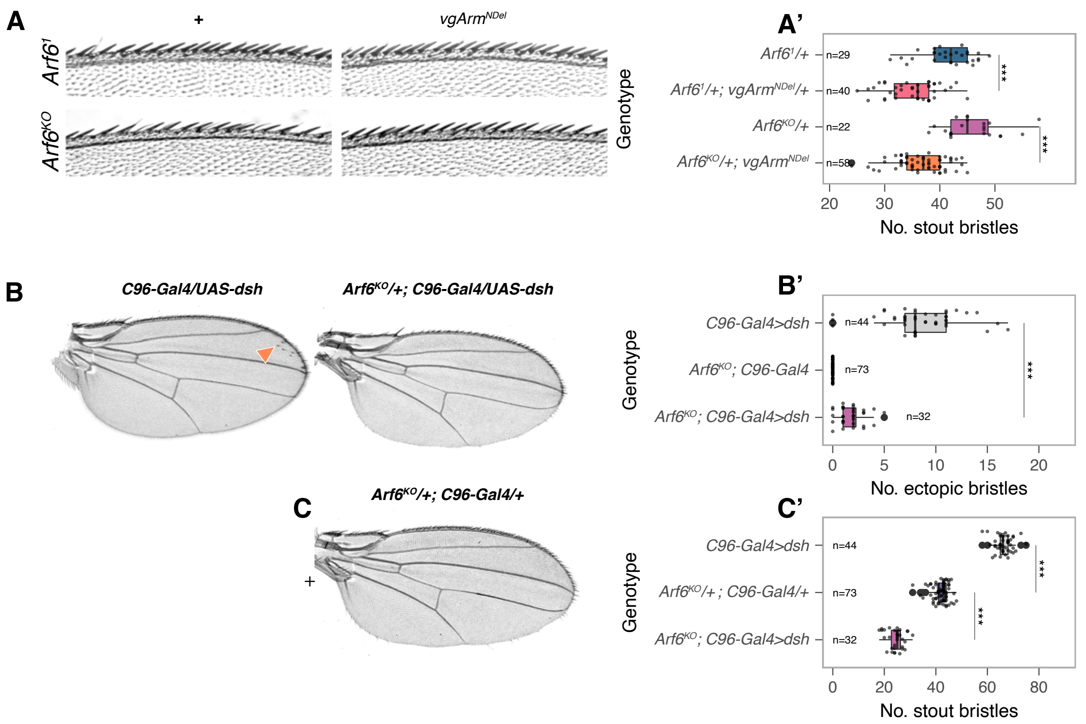

Fig. S5. Wg activation b. vgArm^{Neel} and dsh over-expression enhance the Arf6 phenotype

(A) The loss of stout wing margin bristles in a heterozygous *Arf6* mutant background is enhanced by the expression of vgArm^{NDel} (quantified in **A'**). **(B)** WT *dsh* over-expression triggers high level Wg signalling and the formation of ectopic bristles (closed orange arrowhead). **(B')** These bristles are dominantly suppressed in the *Arf6*^{KO} background. **(C)** Similar to the effect of Arm^{S10} and vgArm^{NDel}, the number of stout mechanosensory bristles is reduced by the over-expression of dsh in an *Arf6*^{KO} background (quantified in **C').**

Biology Open • Supplementary information

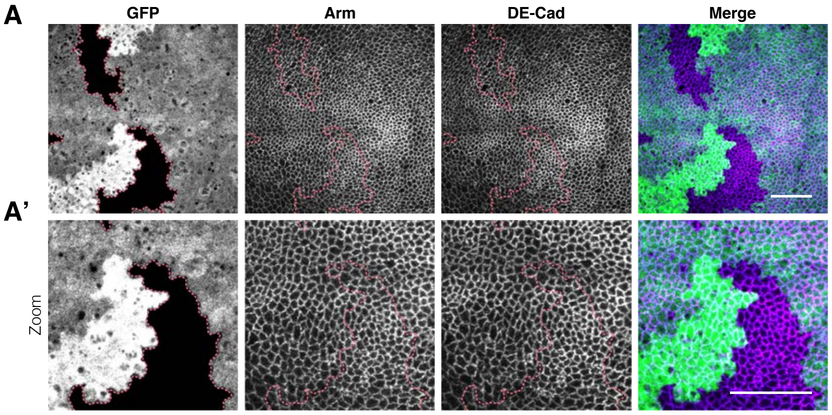

Fig. S6. Arm and DE-Cadherin localisation is not affected in Arf6 mutants

(A) Staining for adherens junction components Arm and DE-Cadherin in Arf6KO clones. (A') A zoom of the *Arf6*^{KO} mutant clone is shown. In the merges, GFP is in green, Arm is in blue and DE-Cad is in magenta. n = 28.

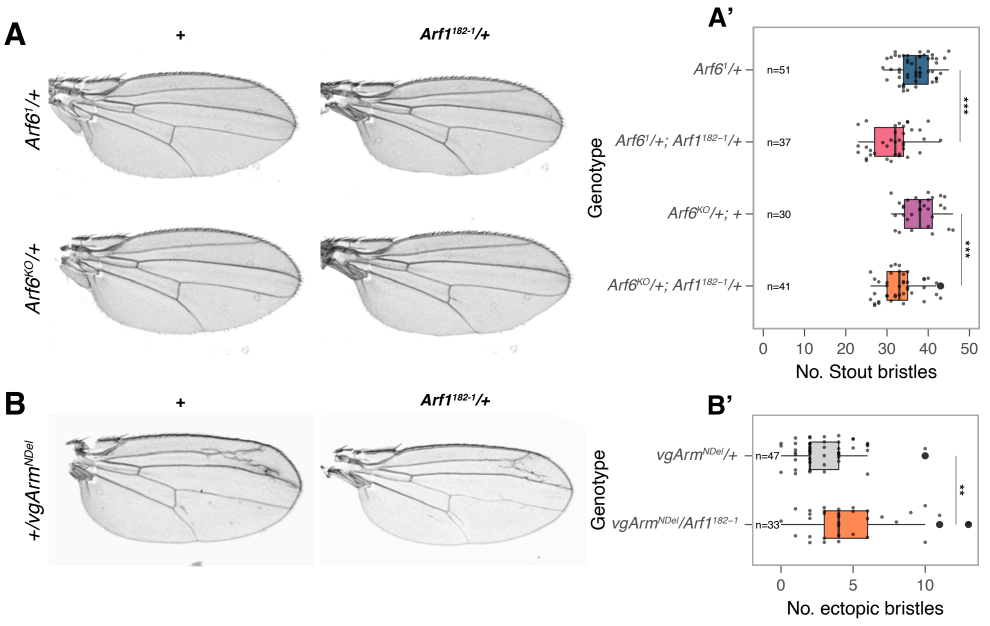

Fig. S7. Arf6 acts independently of Arf1 in Wingless signalling

(A) Consistent with an upstream role of Arf1 in Wg signal transduction (Hemalatha et al., 2016), removing a copy of *arf1* (*arf1*$^{182\text{-}1}$ (West et al., 2017)) in a heterozygous *Arf6*^{KO} mutant background mildly enhances the loss of anterior stout mechanosensory bristles (quantified in **A')**. **(B)** Unlike *Arf6* mutants, *arf1*$^{182\text{-}1}$ does not reduce the number of ectopic bristles induced by vgArm^{NDel} (quantified in **B')**. **)**. This suggests that Arf1 and Arf6 play distinct roles in wing margin patterning.

Biology Open • Supplementary information

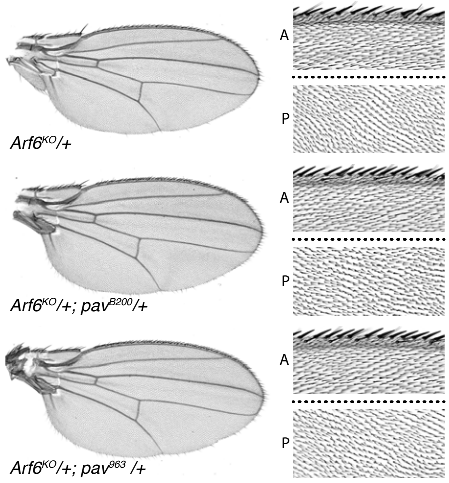

Fig. S8. The genetic interaction between Arf6 and pav does not induce cytokinesis defects

(A) anterior and posterior zooms of wings of the genotypes shown in figure **4A** showing details of trichomes. No multiple wing hair phenotype is present.

Biology Open • Supplementary information

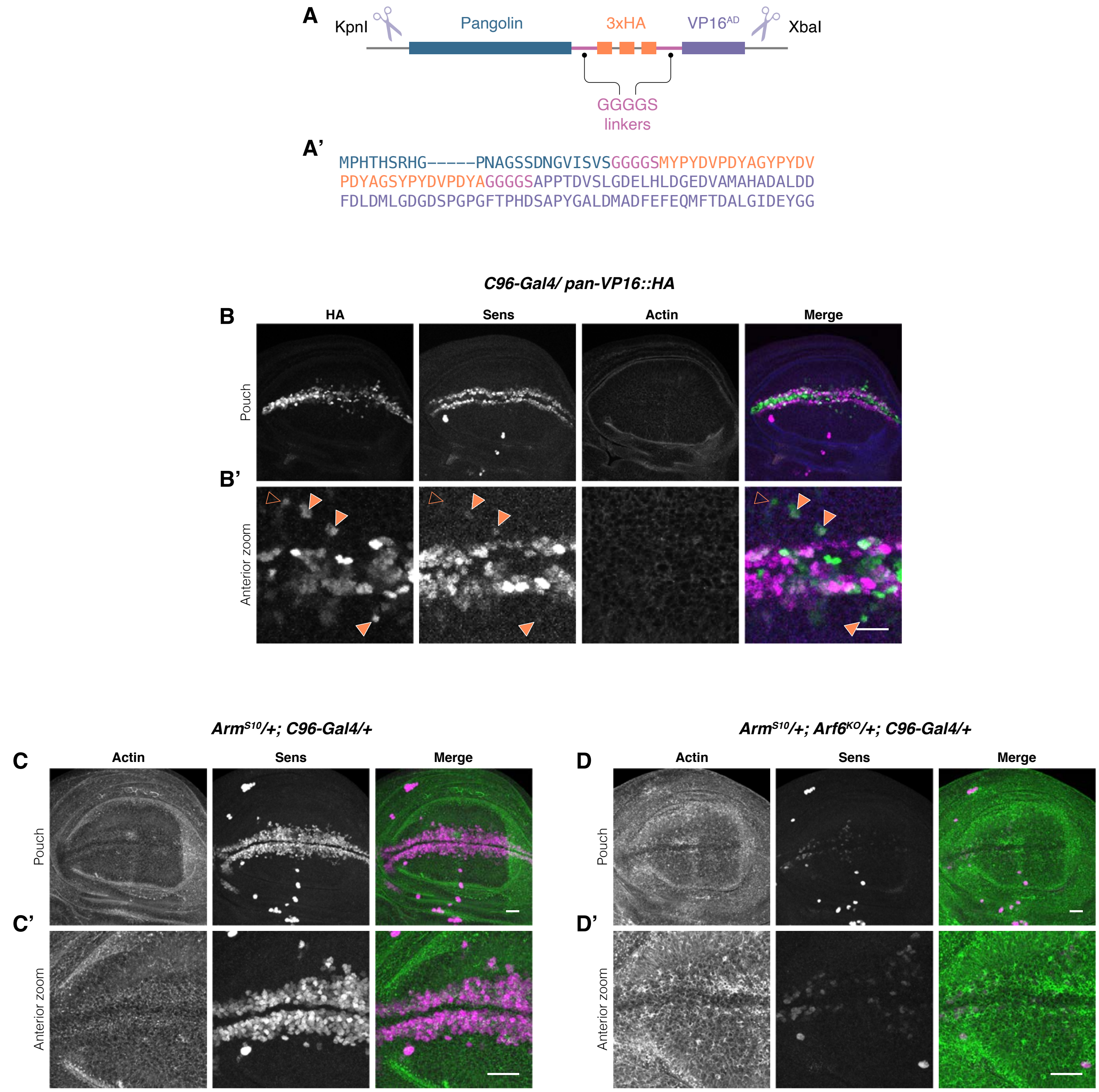

Fig. S9. Structure and activity of Pan-VP16

(A) schematic showing the structure of the pan-VP16 construct. The full-length pan coding sequence (blue) was synthesised with a sequence encoding the activation domain of herpes simplex VP16 (purple). 3xHA tags (orange) were introduced between the two sequences, with sequences encoding GGGGS linkers at either end (pink). The sequence was flanked by 5’ KpnI and 3’ XbaI. **(A’)** Abbreviated primary sequence of the Pan-VP16 construct is presented. The sequence colours

Hemalatha, A., Prabhakara, C. and Mayor, S. (2016). Clathrin-Independent Endocytosis of Wingless via Clic/Geec Pathway is Necessary for Effective Signalling in Drosophila Wing Discs. *Proc. Natl. Acad. Sci.* **110**, 595a.

Huang, J., Zhou, W., Dong, W., Watson, A. M. and Hong, Y. (2009). Directed, efficient, and versatile modifications of the Drosophila genome by genomic engineering. *Proc. Natl. Acad. Sci. U. S. A.* **106**, 8284–8289.

Nolo, R., Abbott, L. A. and Bellen, H. J. (2001). Drosophila Lyra mutations are gain-of-function mutations of senseless. *Genetics* **157**, 307–315.

West, J. J., Zulueta-Coarasa, T., Maier, J. A., Lee, D. M., Bruce, A. E. E., Fernandez-Gonzalez, R. and Harris, T. J. C. (2017). An Actomyosin-Arf-GEF Negative Feedback Loop for Tissue Elongation under Stress. *Curr. Biol.* **27**, 2260-2270.e5.
